# Distinct fecal microbiome between wild and habitat-housed captive polar bears (*Ursus maritimus*): Impacts of captivity and dietary shifts

**DOI:** 10.1371/journal.pone.0311518

**Published:** 2024-11-20

**Authors:** Jing Lu, Renee Petri, Dylan McCart, Amy Baxendell-Young, Stephanie Anne Collins

**Affiliations:** 1 Faculty of Agriculture, Department Animal Science and Aquaculture, Dalhousie University, Truro, NS, Canada; 2 Sherbrooke Research and Development Centre, Agriculture and Agri-Food Canada, Sherbrook, QC, Canada; 3 Churchill Northern Studies Centre, Churchill, MB, Canada; 4 Cochrane Polar Bear Habitat, Cochrane, ON, Canada; Institute for Biological Research, University of Belgrade, SERBIA

## Abstract

Understanding the gut microbiome of polar bears can shed light on the effects of climate change-induced prolonged ice-free seasons on their health and nutritional status as a sentinel species. The fecal microbiome of habitat-housed captive polar bears who had consumed a high protein diet long-term was compared with that of the wild population. Individual differences, season, year and dietary inclusion of a brown seaweed (*Fucus spiralis*; part of the natural diet of wild polar bears), as a representation for nutritional change, were investigated for their effects on the fecal microbiome of captive polar bears. Microbial variations among fecal samples from wild and captive polar bears were investigated using 16s rRNA gene based metataxonomic profiling. The captive bears exhibited more diverse fecal microbiota than wild bears (p<0.05). The difference was due to significantly increased *Firmicutes*, *Campilobacterota* and *Fusobacteriota*, decreased *Actinobacteriota* (p<0.05), and absent *Bdellovibrionota* and *Verrucomicrobiota* in the captive bears. Compared with other factors, individual variation was the main driver of differences in fecal microbial composition in the captive bears. Seaweed consumption did not alter microbial diversity or composition, but this did not rule out dietary influences on the hosts. This is the first study, to the best of our knowledge, comparing the fecal microbiota of captive and wild polar bears and it reveals distinct differences between the two groups, which could result from many factors, including available food sources and the ratio of dietary macronutrients. Our findings provide preliminary insights into climate-change induced dietary shifts in polar bears related to climate-associated habitat change.

## Introduction

Polar bears (*Ursus maritimus*) have a unique preference for a high fat, energy-dense diet, comprised mainly of seals [1,2]. When food is abundant, they purposely target seal blubber (the insulating outer fat layer of a seal) and abandon the rest of the carcass [3,4]. This stems from the substantial energy required to meet their basal metabolic and locomotor demands in the extreme Arctic weather, compared to other mammals with a similar body size [5,6]. Historically, polar bears either prey on seals year-round or rely on body fat stored in winter and spring to survive the ice-free season, regaining lost weight upon returning to sea ice [7].

The current trend of shrinking Arctic sea ice coverage, with an 18% loss in 2020 in comparison to 1982 [8], and the extended ice-free season due to earlier sea ice break-up and later refreeze reduces the area and window of time polar bears can spend on the sea ice platform [9]. Some polar bear populations are compelled to increase land use [10], switching to hunting terrestrial animals. On land, bears are opportunistic feeders and consume a wide range of food items. These include rodents, caribou, fish and marine invertebrates, grasses, marine algae, berries, mushrooms, and anthropogenic waste [11,12]. Onshore bears in the present day have an increased volume of animal items in their diet compared to 40+ years ago (2006–2008 versus 1968–1969), and hunt prey they were previously reported to show little interest in, such as reindeer [7,11,13].

The consequences of transitioning from sea ice to the terrestrial platform are beyond a mere dietary pattern change. With decreased opportunities to feed on high-energy food and reduced energy payoff per prey, polar bears are at a higher risk for energy deficit [5,6], which has already been observed in some populations [5,14]. From a nutritional standpoint, terrestrial diets, higher in lean protein and devoid of marine fat, pose a major shift in dietary macronutrients. As the ice-free season progressively prolongs, some polar bears are forced to consume this low-fat diet for extended periods, potentially exceeding their physiological constrains. Diverging from brown bears 479–343 thousand years B.P., polar bears have undergone tremendous physiological changes and evolved to efficiently metabolize a high fat diet, while rapidly clearing low-density lipoprotein cholesterol from the blood stream to negate cardiovascular diseases associated with this lifestyle [15]. Polar bears have shown a clear preference for a high fat diet, both in the wild and under captivity during ad libitum feeding conditions [4,16–18]. Impacts of dietary macronutrients alteration are often understudied in wild animals such as polar bears, but carry significant implications for their survival, well-being and welfare.

Microbes are a key indicator of host nutritional and health status, as they are involved in various biological process such as fibre digestion, synthesis of short chain fatty acids, amino acids and vitamins, and immune regulation [19–22]. Microbiological ecology of the mammalian gut ecosystem indicates the stability of the gut microbiota is highly dependent on diet and dietary stability in other species [23]. The gut microbiome provides useful insights to evaluate the impact of climate change-driven dietary shifts in this sentinel species. Research on the gut microbiome of polar bears is limited, primarily in wild bears, and based on fecal samples [24–27]. The dominant phyla in fecal samples of polar bears demonstrated by 16S rRNA gene amplicon sequencing are *Firmicutes*, *Proteobacteria*, *Actinobacteria*, and *Bacteroidota*, accounting for over 95% of the total bacteria present in the samples analyzed [25,26].

The gut microbiome of polar bears varies among and within subpopulations [25,26]. Onshore bears exhibit a greater gut microbiome diversity than bears living offshore [26], which is likely due to a more varied diet [12]. These findings raise concerns about the implications of climate change-driven habitat and dietary shifts on the gut and overall health of this species. The present study sought to explore these impacts by comparing the gut microbiome of captive polar bears with that of the wild population. Our investigation, however, extends beyond captivity and habitat change. The four captive bears in our study, Henry, Ganuk, Inukshuk and Eddy, were housed at the Cochrane Polar Bear Habitat (Cochrane, ON, Canada) with access to outdoor enclosures of up to 21 acres in a natural landscape. The captive habitat lifestyle represents the repercussions of a scenario where Arctic sea ice continues to decline to a point that it fails to refreeze during the winter months, forcing bears to remain onshore and feed on a low-fat diet permanently. Through this captive habitat model, we aim to understand the implications of shifted dietary patterns and macronutrients on polar bear gut microbiome, which is absent in the existing literature. By understanding the difference between captive and wild polar bears fecal microbiome, we can develop a non-invasive tool to assess the nutritional status of the wild population, as well as to formulate a better captive diet.

In multiple reports including field observations, stomach content examination, and scat analysis, seaweed such as *Fucus spiralis*, has been identified as a dietary component of both onshore and offshore polar bears and they actively seek out seaweed even when food is abundant [11,12,16]. It is curious that an animal who typically feeds on energy-dense food would purposely seek out and consume a low energy density seaweed. A logical speculation would be that consuming seaweed provides biological benefits to polar bears. In mammals, it is suggested that seaweed promotes gut health through its interaction with the gut microbiome [28]. However, the impact of seaweed on the gut microbiome of polar bears is unclear. Therefore, we introduced seaweed collected from the environment of wild polar bears and fed it to captive polar bears, aiming to restore their gut microbiome to resemble that of wild bears, along with exploring other factors, such as how individual differences, seasonal and annual changes might contribute to shifts in their fecal microbiome.

## Results

There was a total of 17,732,593 raw sequences and after running quality control, 6,356,481 sequences were retained, averaging 42,096 reads per sample. These reads were taken to R for further analysis. In R, after removal of ASVs with less than 10 counts across all samples, and contaminants including mitochondria and chloroplasts, there was a total of 6,146,237 high-quality sequences remaining for statistical analysis. The threshold for sample inclusion was set at a minimum of 8,164 reads, leading to the exclusion of four samples (Henry-day28-2020, Henry-day7-2020, Henry-April-2018, and Ganuk-Nov-2019) from the original 151. Among the 147 remaining samples, 12 were from the wild population and 135 were from the captive polar bears. All samples had a Good’s coverage index of over 0.99.

### Captive versus wild polar bear fecal microbiome

**Fig 1:**
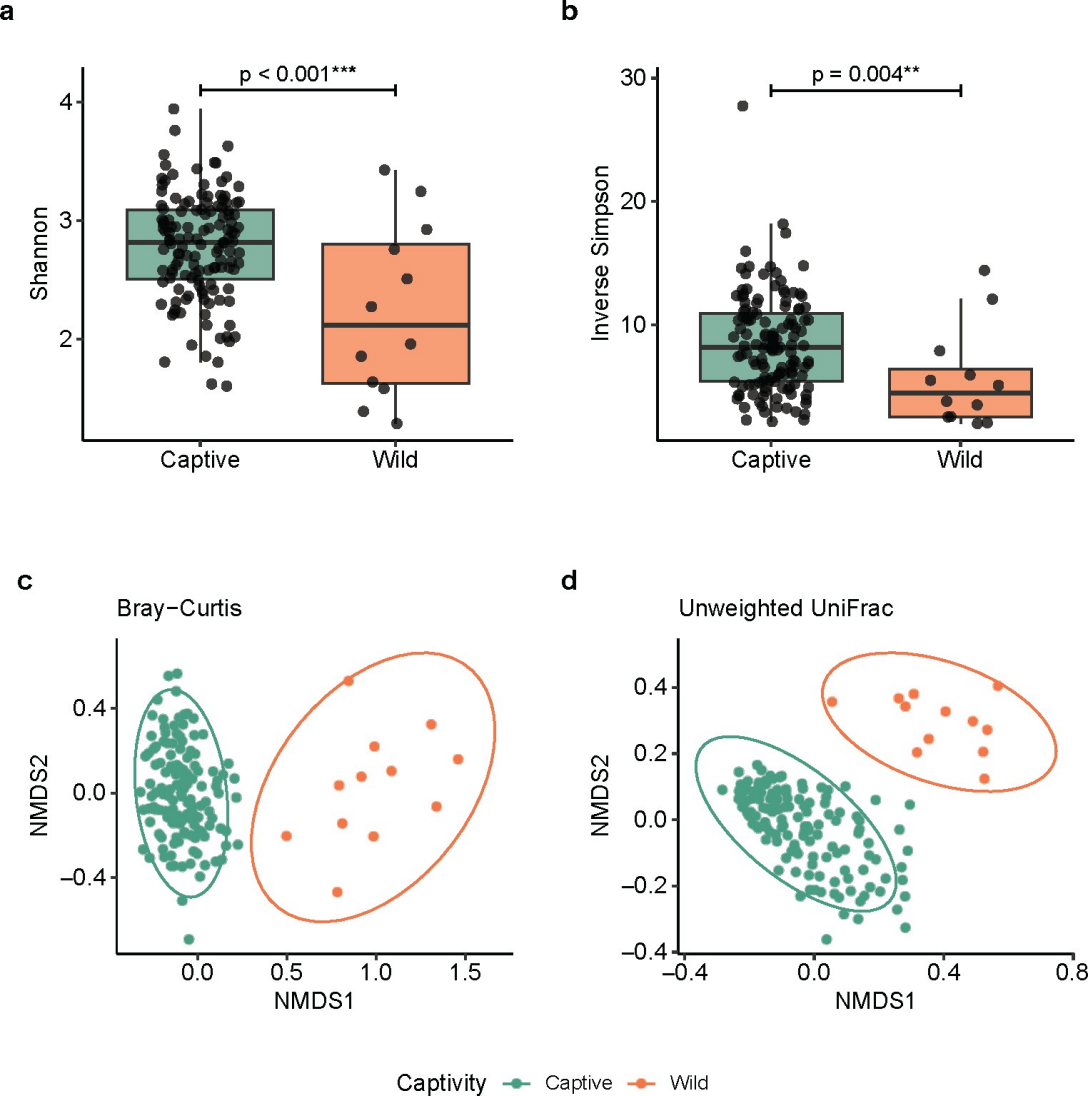
Alpha and beta diversity of the fecal microbiome of captive and wild polar bears: (**a)** Shannon diversity index (p < 0.001); (**b)** Inverse Simpson diversity index (p < 0.05); (**c)** Bray-Curtis distance matrix in a NMDS plot. Stress value = 0.191, PERMANOVA p < 0.001, homogeneity of group dispersions for PERMANOVA test was violated (p = 0.002). ANOSIM p < 0.001, R = 0.779; (**d)** Unweighted UniFrac distance matrix ordinated in a NMDS plot, Stress value = 0.156. PERMANOVA p < 0.001, homogeneity of group dispersions was violated (p < 0.001). ANOSIM p < 0.001, R = 0.908.

For alpha diversity, both Shannon and inverse Simpson indices were significantly higher in the captive population compared to the wild polar bears (Fig 1A and 1B). The observed ASVs,Chao1 and Faith’s phylogenetic diversity remained similar between the two populations (S1 Fig). For beta diversity, the two populations showed a clear separation, as demonstrated by both Bray-Curtis and unweighted UniFrac distance matrices in non-metric multidimensional scaling (NMDS) plots (Fig 1C and 1D). This was further confirmed by both permutational multivariate analysis of variance (PERMANOVA) and analysis of similarities (ANOSIM) analyses (p < 0.05). It should be noted that the homogeneity of dispersions was violated for PERMANOVA, likely due to unbalance sample numbers between the wild and captive populations (12 vs 135 samples).

We further conducted direct comparisons of the fecal microbiome alpha (S1 Table) and beta (S2 Fig and S2 Table) diversity between the captive bear samples from Cochrane and wild bear samples from Churchill and Fort Severn. The microbiome of the wild bear samples from both locations clustered together, separating them from the captive bears samples from Cochrane (S2 Fig). This observation was statistically supported by PERMANOVA, ANOSIM, and pairwise PERMANOVA tests (p < 0.05) (S2 Table).

**Fig 2:**
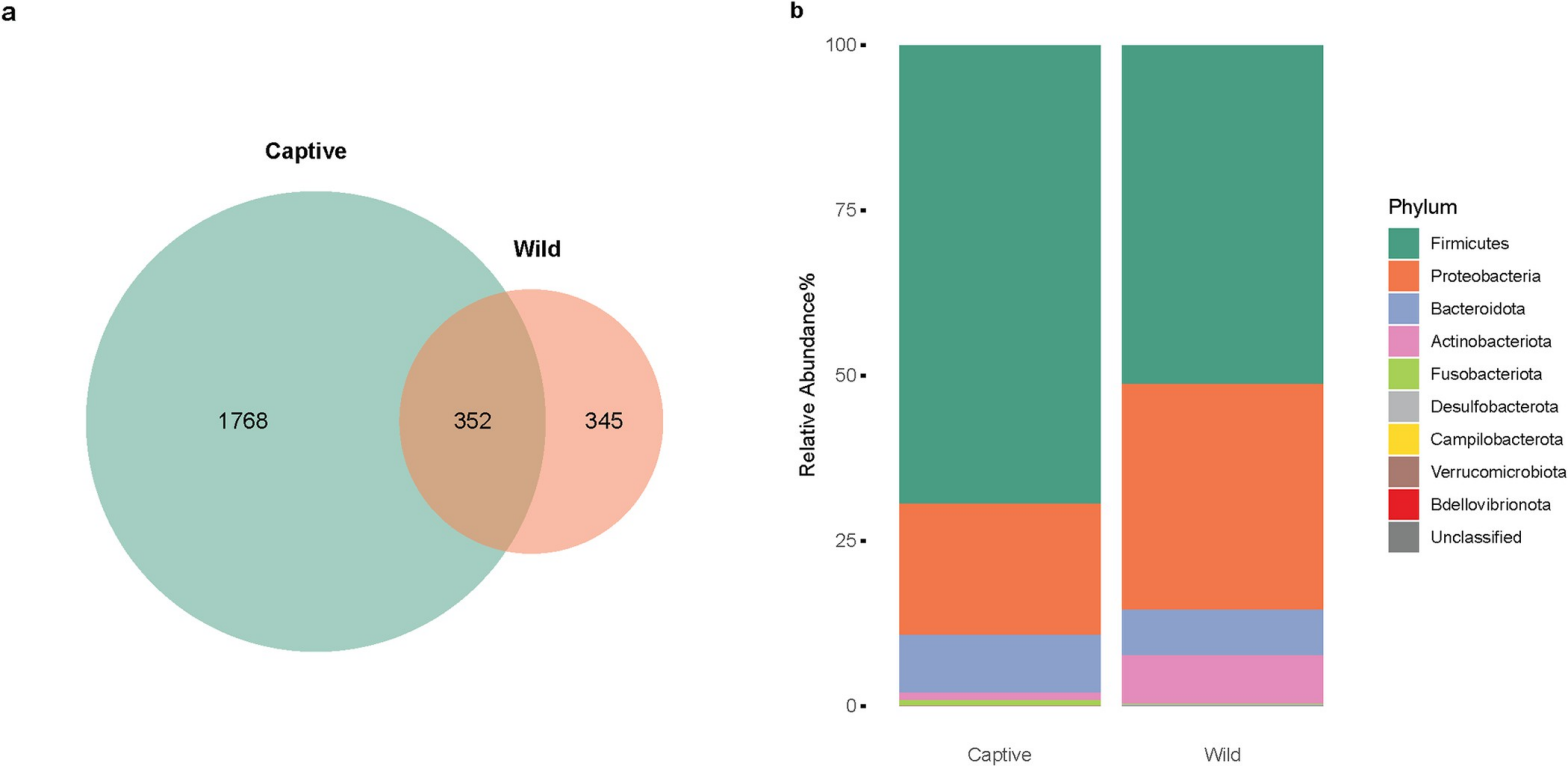
(a) Comparison of unique and shared amplicon sequence variants (ASVs) between the captive and wild polar bear populations demonstrated in a Venn diagram. (b) Relative abundance of both captive and wild polar bear fecal microbiome at the phylum level.

**Fig 3:**
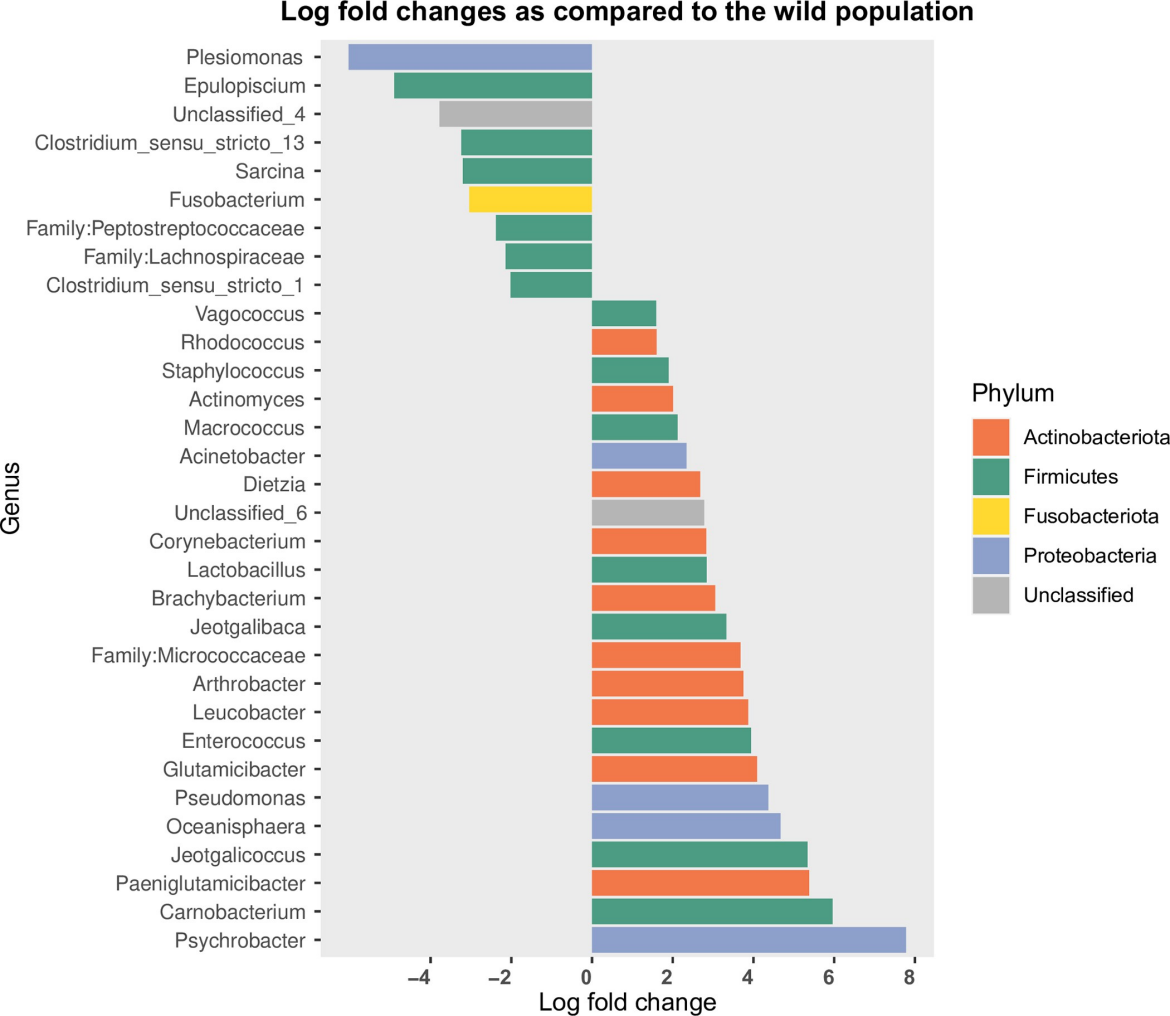
Fecal microbial composition analysis with ANCOM-BC at the genus level: A comparison between the captive and wild populations.

The captive and wild populations had a total of 2,120 and 697 unique ASVs, respectively, with 352 being shared between the two populations (Fig 2A). At the phylum level, *Firmicutes*, *Proteobacteria*, *Bacteroidota* and *Actinobacteriota* were the most abundant phyla, accounting for over 95% in both populations (Fig 2B). However, seven of nine classified phyla, including *Firmicutes*, *Proteobacteria*, *Actinobacteriota*, *Fusobacteriota*, *Desulfobacterota*, *Campilobacterota*, *Verrucomicrobiota*, were significantly different between the two populations. Compared to the wild population, the captive bears had a significantly higher relative abundance of *Firmicutes*, *Fusobacteriota*, *Campilobacterota* and unclassified bacteria, as well as lower *Proteobacteria* and *Actinobacteriota* populations (p < 0.05) (S3 Table). Both *Bdellovibrionota* and *Verrucomicrobiota* were unique to the wild bears. At the family level, the captive population showed a one- to eight-log fold increase of 21 families and a one- to four- log fold decrease of nine families, compared with the wild population, based on the analysis of compositions of microbiomes with bias correction (ANCOM-BC; S3 Fig). At the genus level, the fecal microbiome of the captive population had between one- to eight- log fold higher occurrence of 23 genera, and a one- to five- log lower occurrence of nine genera, compared to the wild population (Fig 3).

### Fecal microbiome within the captive population

To investigate variation among captive polar bear populations in relation to individual differences (four captive bears: Inukshuk, Ganuk, Henry, and Eddy), seasonal changes (spring, summer, fall, and winter), and year of collection (2018, 2019, and 2020), monthly fecal microbiome data were used. All four bears were male and of average body condition, therefore body condition was not considered as a factor. Fecal samples collected during or one month after the seaweed feeding trials were excluded in this subset to eliminate variation associated with the experimental diet. As a result, this dataset contained a total of 93 samples.

Within the captive population, individual differences were significant, while season and year had minimal influence on the fecal microbiome (p > 0.05). Observed ASVs, Chao1 and Faith’s phylogenetic diversity indices were significantly higher in Ganuk when compared to Inukshuk (Table 1), and those of Eddy were intermediate. The species evenness indices, Shannon and inverse Simpson, were similar among all bears (p > 0.05). All five diversity indices, including observed ASVs, Chao1, Shannon, inverse Simpson, and Faith’s phylogenetic diversity were similar among seasons and years (p > 0.05), except that captive bears in the fall months had a significantly lower Faith’s phylogenetic diversity index than in the spring months (p < 0.05; Table 1).

**Table 1:** Alpha diversity of fecal microbiome of captive polar bears housed at Cochrane habitat.

	**By individual differences**				** **
	Ganuk	Henry	Inukshuk	Eddy	ANOVA p-value
Observed ASVs	146.9 ± 38.4 ^a^	141.3 ± 42.7 ^ab^	117.8 ± 36.8 ^b^	132.1 ± 39.2 ^ab^	0.049*
Chao1	205.6 ± 60.3 ^a^	186.4 ± 52.0 ^ab^	160.1 ± 53.0 ^b^	177.5 ± 57.6 ^ab^	0.034*
Shannon	2.9 ± 0.5	2.8 ± 0.4	2.7 ± 0.5	2.5 ± 0.5	0.055
Inverse Simpson	9.0± 3.1	8.8 ± 3.1	8.0 ± 3.8	6.6 ± 4.4	0.169
Faith’s phylogenetic diversity	20.0 ± 5.1 ^a^	19.3 ± 5.7 ^a^	15.5 ± 4.2 ^b^	17.9 ± 5.3 ^ab^	0.012**
	**By season**				** **
	Spring	Summer	Fall	Winter	ANOVA p-value
Observed ASVs	143.1 ± 28.3	132.1 ± 40.2	121.9 ± 33.2	144.1 ± 53.7	0.238
Chao1	198.6 ± 46.1	183.5 ± 56.3	166.8 ± 56.5	184.3 ± 68.7	0.374
Shannon	2.7 ± 0.5	2.7 ± 0.4	2.8 ± 0.4	2.8± 0.5	0.916
Inverse Simpson	7.0 ± 2.7	8.9 ± 4.0	9.0 ± 3.4	7.9 ± 3.7	0.201
Faith’s phylogenetic diversity	20.1 ± 3.7 ^a^	18.1 ± 5.2 ^ab^	15.6 ± 4.8 ^b^	19.1 ± 6.4 ^ab^	0.040*
	**By year**				
	2018	2019	2020		ANOVA p-value
Observed ASVs	129.1 ± 37.4	131.3 ± 42.1	143.4 ± 40.7		0.334
Chao1	184.6 ± 55.0	176.3 ± 58.8	189.5 ± 59.0		0.638
Shannon	2.7 ± 0.4	2.8 ± 0.5	2.7 ± 0.5		0.564
Inverse Simpson	7.9 ± 3.3	8.9 ± 3.7	7.9 ± 3.7		0.424
Faith’s phylogenetic diversity	16.8 ± 4.7	18.0 ± 5.4	19.6 ± 5.4		0.127

Square root of inverse Simpson was used to achieve normal distribution of the residuals.

Mean ± SD. Different superscripts in the same row indicate statistical significance (p < 0.05).

**Fig 4:**
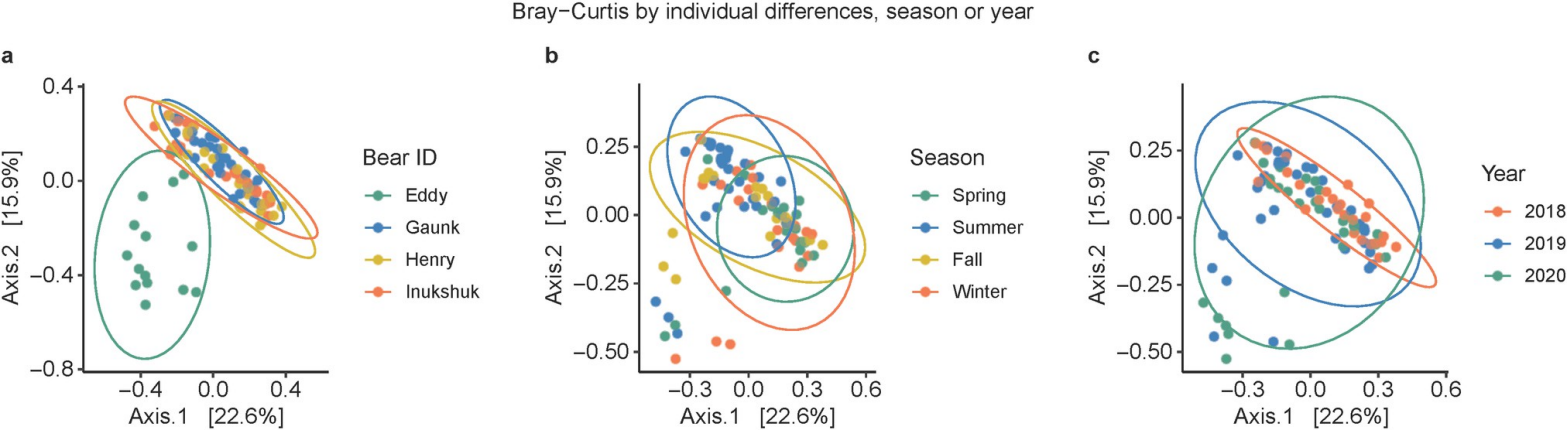
Beta diversity of captive polar bear fecal microbiome demonstrated by Bray-Curtis distance matrix in PCoA plots: Effects of **a)** individual differences, **b)** season, **c)** year.

Similar to alpha diversity, season and year had minimal impacts on beta diversity, whereas there was a clear separation among individual bears as shown by the Bray-Curtis distance matrix ordinated in a principal coordinates analysis (PCoA) plot (Fig 4). Bear Eddy’s fecal microbiome was significantly distinct from the other three captive bears. This observation was confirmed by both PERMANOVA and ANOSIM analyses (Table 2), which indicated that individual differences accounted for most of the variation (p < 0.001, R^2^ = 0.201). While season and year were also statistically significant, the relatively small R^2^ values (0.094 and 0.056, respectively) indicated that these two factors contributed little to beta diversity.

**Table 2:** PERMANOVA) and ANOSIM by individual differences, season, and year in the captive polar bear fecal microbiome.

	PERMANOVA					ANOSIM	
	p-value	R^2^	Homogeneity of group dispersions			p-value	R
By individual differences	<0.001	0.201	0.424			<0.001	0.319
By season	<0.001	0.094	0.643			<0.001	0.110
By year	<0.001	0.056	0.050			<0.009	0.055

The fecal microbial composition of captive bears differed significantly among individual bears. At the phylum level, Eddy showed a significantly different profile compared to the other three bears, with decreased relative abundances of *Firmicutes* and *Proteobacteria*, and a nearly 10-fold increase in *Bacteroidota* (Table 3). Inukshuk showed a higher abundance of *Firmicutes* compared to Ganuk and Henry, as well as significantly lower abundances of *Proteobacteria*, *Bacteroidota*, and *Fusobacteriota* compared to Ganuk (p < 0.05). Season also influenced fecal microbial composition, with differences primarily observed between winter and the other seasons (Table 3).

**Table 3:** Relative abundance (%) of at the phylum level among captive bears: Variations by individual, season, and year.

**Relative abundance (%)**	**By individual differences**					
	Eddy	Ganuk	Henry	Inukshuk		Kruskal-Wallis p-value
*Firmicutes*	51.7±15.9 ^a^	66.5±15.7 ^b^	70.9±11.9 ^b^	78.5±13.6 ^d^		< 0.01**
*Proteobacteria*	7.1 ± 6.2 ^a^	28.1 ± 17.7 ^b^	23.5 ± 13.7 ^bc^	18.2 ± 11.8 ^c^		< 0.01**
*Bacteroidota*	38.9 ± 17.6 ^a^	3.5 ± 4.3 ^b^	2.9 ± 4.0 ^bc^	2.6 ± 6.9 ^c^		< 0.01**
*Actinobacteriota*	1.24 ± 2.46	0.75 ± 1.44	1.32 ± 2.15	0.29 ± 0.45		0.447
*Fusobacteriota*	1.00 ± 0.90 ^a^	1.01 ± 1.75 ^ab^	1.33 ± 4.67 ^ab^	0.43 ± 0.93 ^b^		< 0.01**
*Desulfobacterota*	0.020 ± 0.034	0.087 ± 0.223	0.060 ± 0.110	0.018 ± 0.065		0.311
*Campilobacterota*	0.057 ± 0.093	0.015 ± 0.027	0.030 ± 0.095	0.022 ± 0.039		0.46
Unclassified bacteria	0.009 ± 0.011	0.012 ± 0.022	0.010 ± 0.015	0.007 ± 0.017		0.662
	**By season**					
	Spring	Summer	Fall	Winter		Kruskal-Wallis p-value
*Firmicutes*	72.0 ± 17.7	62.3 ± 15.5	72.5 ± 13.4	71.7 ± 17.4		0.054
*Proteobacteria*	17.5 ± 15.2 ^ab^	28.4 ± 15.4 ^a^	18.8 ± 11.9 ^ab^	14.7 ± 14.3 ^b^		< 0.01**
*Bacteroidota*	8.22 ± 13.9	7.21 ± 15.2	7.92 ± 13.7	11.4 ± 19.0		0.257
*Actinobacteriota*	1.54 ± 2.26 ^a^	0.55 ± 1.21^ab^	0.29 ± 0.88 ^b^	1.20 ± 2.15 ^a^		< 0.01**
*Fusobacteriota*	0.65 ± 0.21	1.50 ± 1.90	0.48 ± 0.26	0.80 ± 0.34		0.859
*Desulfobacterota*	0.028 ± 0.008 ^a^	0.012 ± 0.007 ^a^	0.041 ± 0.021 ^ab^	0.142 ± 0.049 ^b^		< 0.01**
*Campilobacterota*	0.024 ± 0.007 ^ab^	0.020 ± 0.009 ^ab^	0.009 ± 0.003 ^a^	0.060 ± 0.013 ^b^		0.014*
Unclassified bacteria	0.009 ± 0.004	0.009 ± 0.002	0.014 ± 0.005	0.008 ± 0.002		0.585
	**By year**					
	2018	2019	2020			Kruskal-Wallis p-value
*Firmicutes*	71.8 ± 14.5	71.8 ± 16.1	62.5 ± 17.0			0.041 ^1^
*Proteobacteria*	23.5 ± 15.2	18.1 ± 14.6	21.9 ± 16.0			0.301
*Bacteroidota*	3.6 ± 6.9	8.7 ± 14.5	12.5 ± 20.2			0.333
*Actinobacteriota*	0.57 ± 1.05	0.62 ± 1.12	1.38 ± 2.50			0.1
*Fusobacteriota*	0.56 ± 1.04	0.67 ± 1.29	1.58 ± 4.32			0.338
*Desulfobacterota*	0.025 ± 0.067	0.057 ± 0.132	0.065 ± 0.191			0.809
*Campilobacterota*	0.012 ± 0.026	0.017 ± 0.039	0.052 ± 0.103			0.475
Unclassified bacteria	0.005 ± 0.011 ^a^	0.005 ± 0.010 ^a^	0.018 ± 0.024 ^b^			0.001**

Mean ± SD. Different superscripts in the same row indicate statistical significance.

^1^ Although the p-value was less than 0.05, the post hoc analysis using the Dunn test did not detect any significant differences.

Since Eddy’s fecal microbiome significantly differed from those of the other three captive bears, we conducted additional alpha (S4 Table) and beta diversity (S4 Fig and S5 Table) analyses to explore variations within captive bears after excluding Eddy. As expected, the impact of individual differences on beta diversity decreased after the exclusion of Eddy, with R^2^ decreased from 0.201 (Table 2) to 0.059 (S5 Table). The effect of season became slightly more pronounced, with the R^2^ increased from 0.094 (Table 2) to 0.126 (S5 Table).

### Macroalgae feeding trials with Ganuk, Henry and Inukshuk

**Fig 5:**
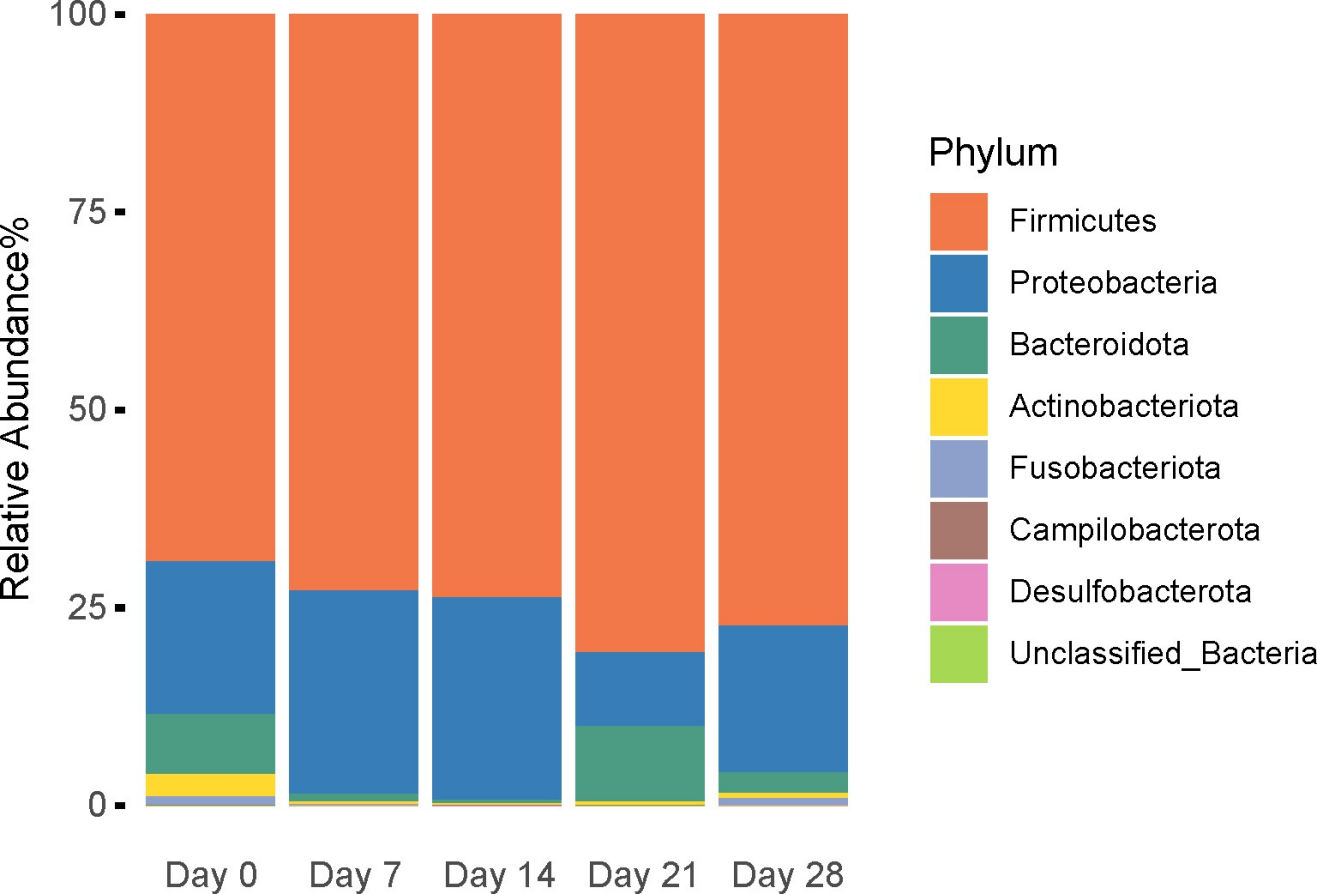
Relative abundance at the phylum level of captive polar bears fed seaweed. Kruskal-Wallis tests conducted for each phylum showed no significant differences with all p > 0.05.

Eddy was excluded from this set of analyses due to his significantly different fecal microbiome from the other three bears. Using ANOVA with repeated measures and trial (trial 2019 vs 2020) as a blocking factor, we found that all five alpha diversity indices were similar (p > 0.05) at each sampling point throughout the 28-day trial except for the Shannon index (S6 Table). The Shannon index on day 14 was significantly lower than that on day 7, although this difference disappeared in the subsequent periods (S6 Table). The blocking factor was not significant (p > 0.05). No clear separation was observed in PCoA and NMDS plots using unweighted, weighted UniFrac and Bray-Curtis distance matrices (S5 Fig), and this observation was confirmed by PERMANOVA and ANOSIM analyses (all p > 0.05, S7 Table). Seaweed did not change the relative abundance of any phylum at different sampling points (p > 0.05, Fig 5). Additionally, ANCOM-BC was run at the genus level to compare the fecal microbiome on days 7, 14, 21, and 28 with that of day 0 (S6 Fig). No significant difference was detected except that genus *Lactococcus* had a 2.31-log fold increase on day 7 compared to day 0.

## Discussion

The lifestyle of captive polar bears in this study simulated the possible habitat and lifestyle changes of this species under climate change, that is, with continued decline of Arctic sea ice, most polar bears, including those from the Hudson Bay region, will likely remain onshore permanently with no access to their preferred marine mammal-based diet. Through comparisons with the fecal microbiome of the wild population, we confirmed that captive polar bears housed in the polar bear habitat had a more diverse fecal microbiome that was very different from the wild population. This study also investigated variations in the fecal microbiome within the captive population in relationship to factors including individual differences, seasonal changes, annual variations, as well as the importance of a dietary component, brown seaweed, which was part of their natural diet in the wild. To the best of our knowledge, this study demonstrates the first comparison of the fecal microbiome of captive and wild polar bears and sheds light on the potential impacts of climate change on polar bears and their gut microbiome.

### Comparison of fecal microbiome of captive and wild polar bears

Our results agree with most studies conducted in other species (carnivores: wolf, African wild dog and leopard, primates such as monkeys and howlers, herbivores: Andean bears) that the gut microbiome of wild populations differs from their captive counterparts [27,29]. Captive polar bears in this study had a more diverse fecal microbiome compared to the wild onshore bears. Integrating our results with previous polar bear studies in which onshore population demonstrated a more diverse fecal microbiome than the offshore population, the fecal microbiome diversity of polar bears increased progressively, with the lowest diversity in offshore bears, intermediate diversity in onshore bears and the highest diversity in the captive habitat setting [25,26]. One explanation may be that dietary options are more abundant onshore and in the captive environment observed, compared to offshore. In our study, the captive bears were subjected to a diet similar to that of onshore polar bears, which included lean proteins from various meats and fish, a small amount of seal oil, and a range of in-season produce that wild bears might not have access to. Additionally, the four captive polar bears were exposed to lakes, terrestrial plants, and other wildlife when being rotated among five enclosures ranging from 0.5 to 21 acres at the habitat.

Another explanation for the differences observed in the fecal microbiome of captive and wild polar bears could be due to different macronutrients. Although the diets of the wild polar bears sampled were unknown, the microbiome signature of the captive bears, when compared to these wild ones, mirrors the pattern observed in high protein versus high fat fed mice [30]. Specifically, no difference was observed in microbial species richness, but species evenness was significantly higher in mice fed a high protein diet. Additionally, as protein level increased, the *Firmicutes* to *Bacteroidetes* ratio decreased, as well as the relative abundance of *Verrucomicrobiota* [30]. Different from onshore wild polar bears that are still able to switch back to a high fat diet when they return to sea ice, the captive bears in our study have been on the high-protein diet for most, if not all their lives.

The long-term effects of a high protein diet on this species have not been extensively studied. However, kidney disease has been the primary cause of death in polar bears housed in conventional zoos across the US between 2015 and 2020. Thirty-seven percent (7 out of 19 bears) of the captive polar bears in these zoos have died of kidney disease, which surpassed the combined mortality rate caused by liver disease and cancer at 32% (6 out of 19 bears) [17]. Combining this fact with polar bears’ biological instinct to choose a high fat diet over a high protein diet, it is possible that this species have evolved to metabolize higher levels of dietary protein only on a short-time basis, primarily to complement their fat intake during specific events such as annual migration between sea ice and shore. However, polar bears do not appear to have the capacity to function on high protein diets over the long term after they diverged from other carnivores 47.5 million years ago [31]. While the altered microbiome in the captive bears in the present study alone is insufficient to predict the consequences of long-term protein overconsumption, it is clear that the protein levels in the diets of captive polar bears need to be reconsidered.

The comparison between wild and captive bears in our study could have some method-related biases; samples from the captive population were stored in RNAlater^TM^ and kept at -20°C, as this preserves the quality of DNA for long-term storage. However, it was not possible to follow the same collection procedure at that time in the field. This variation in procedure could have impacted the total number of high-quality sequences in the wild samples. Further limitations are related to the fecal samples from wild bears, which include a small sample size, unknown host conditions, as well as sample quality based on the age of the feces and environmental temperature. These factors could potentially create biases in the general community structure, although these biases were not evident in the phylogenetic comparisons. Future studies should use larger sample sizes and optimized methods to examine both gut microbiome and host health-related metabolites to provide a more comprehensive understanding of the potential outcomes of habitat changes.

Regardless of the limitations of the fecal samples from wild onshore bears discussed above, the number of ASVs identified and the microbiome composition on the phylum level of our wild samples were comparable to a previous study using similar sequencing and bioinformatics methods [25]. In our study, a total of 697 ASVs were identified in the fecal samples of wild bears from the Hudson Bay shore area. This number is comparable to the 742 ASVs identified in the East Greenland wild population and slightly fewer than the 940 ASVs observed in the Southern Beaufort Sea population [25]. Consistent with previous studies, the four dominant phyla, including *Firmicutes*, *Proteobacteria*, *Bacteroidota*, and *Actinobacteriota*, accounted for over 95% of the total phyla in the fecal microbiome [25,26]. At the genus level, *Psychrobacter* (family *Moraxellaceae*) was the most enriched genus in the Cochrane captive bears, showing a significant 7.78 log fold increase compared to the wild bears. This finding is consistent with Watson et al. (2019) [26], who reported a 6.78 log fold increase in the family *Moraxellaceae* among the Southern Beaufort Sea onshore bears. These bears have greater land access and diets richer in terrestrial protein and lower in seal fat, compared to the offshore subpopulation. Additionally, other genera such as *Enterococcus*, *Brachybacterium*, *Lactobacillus*, *Corynebacterium*, *Actinomyces*, and *Staphylococcus* also exhibited one- to four- log fold increases in the Cochrane captive bears. Interestingly, these same genera showed comparable increases in the East Greenland bears compared to those in the Southern Beaufort Sea, although the former have greater access to seals than the latter. This unexpected pattern suggests that factors beyond diet play important roles in shaping the microbiome across polar bear populations.

### Fecal microbiome variation within the captive population

Among the three factors examined, individual differences stood out as the main driver, while seasonal changes and year had less of an impact on the fecal microbiome of captive polar bears. Host condition, including sex and body condition, was not considered as all captive polar bears were male and in good body condition. A distinctive case was Eddy, whose fecal microbial diversity significantly deviated from that of the other three captive bears. Eddy had been a captive bear living in a conventional zoo environment prior to being temporarily relocated to the captive habitat. Eddy’s history of conventional zoo lifestyle may have been a factor in his less diverse fecal microbiome. This observation aligns with previous studies that animals housed in conventional zoos usually harbor a less diverse gut microbial community, attributed to their dietary structure [32,33]. Another explanation could be Eddy’s older age compared to Ganuk and Henry, as it is widely recognized that aging alters gut microbiome [34], which could also explain Inukshuk’s lower microbial species richness (observed ASVs, Chao1 and Faith’s phylogenetic; p <0.05) compared to Ganuk.

The impact of season on the fecal microbiome of captive bears in our study agrees with a previous study on wild polar bears which found that Faith’s phylogenetic diversity was the only diversity index changed between spring and fall months, whereas both Shannon and inverse Simpson indices remained unchanged across all seasons [26]. Faith’s phylogenetic diversity is based on the presence or absence of species, while Shannon and inverse Simpson indices also take species evenness into consideration [35]. Our results therefore imply that bacteria species evenness was not altered among seasons, however, bacterial richness might differ among different seasons.

In our study, fecal samples collected from the captive polar bears in fall had a significantly lower Faith’s phylogenetic diversity compared to those collected in spring, which contradicts the results of a previous study where Faith’s phylogenetic diversity was significantly higher in polar bears captured in fall than in spring [26]. Seasonal shifts in gut microbiome have been demonstrated in other animals such as brown bears, and these shifts are highly likely associated with dietary shifts [36]. We speculate that the shift observed in our study was also diet-related.

### Seaweed in the captive polar bear diets

The two seaweed feeding trials were conducted to gain a better understanding of the dietary impact of seaweed on the gut microbiome of polar bears. As Eddy had a significantly different prior history and fecal microbiome than the other three bears, he was removed from the seaweed feeding trial data analysis. Our findings suggest that including seaweed in the diet of captive polar bears had minimal effects on their fecal microbiome. Although this result was unexpected, it can be explained by the fact that captive polar bears in our study had already been exposed to various vegetation as part of their regular habitat diets. Another explanation for the minimal impacts of seaweed being added to the diets of the polar bears could be related to their evolutionary capacity to metabolize seaweed, as evidence indicates that seaweed is a regular component of the wild polar bear diet. Previous studies have shown that switching from a low fat, fiber rich diet to a high fat, low fiber "Western" diet can shift the gut microbial community (beta diversity) in mice and humans after one day, and the microbial community stabilized after a week [37,38]. Although we anticipated that 28 days would be sufficient for the effects of seaweed to manifest, due to the relatively low dietary inclusion rate, it may have been beneficial to feed this diet for a longer duration.

## Conclusion

Understanding the dietary patterns of polar bears and how they are affected by climate change-mediated sea ice decline, as well as the subsequent impacts on gut health through host-microbiota interactions, is critical for assessing the nutritional and health status of these wild populations, promoting species conservation, and evaluating the stability of the Arctic marine ecosystem. Our findings demonstrate significant differences in the fecal microbiome between captive and wild polar bear populations, likely due to habitat changes and dietary shifts, particularly in macronutrient composition. This highlights the potential consequences of diminishing sea ice on this iconic, yet vulnerable species. Within the captive population, the impacts of individual differences were more pronounced than season or year, or the addition of a dietary item, brown seaweed. We recommend future studies to compare the impacts of different levels of dietary fat, protein and carbohydrates on polar bears in consideration of species conservation and animal care practices.

## Materials and methods

Animals used in the present study were cared for in accordance with the policies and guidelines of the Canadian Council on Animal Care and the requirements of the relevant international, federal, provincial, and municipal legislation. The trials conducted with habitat-housed captive polar bears and sample collected from these captive bears were approved by the animal care committee of the habitat.

### Wild onshore polar bear fecal sample collection

Fecal samples from wild polar bears were collected at two locations within the Hudson Bay area in Canada: Churchill, MB, and Fort Severn, ON. One of the two locations, D20 in Churchill, is a temporary holding facility for wild polar bears that have roamed into the local community. Bears are held at this facility until they can be released back to the wild when the sea ice refreezes in the Hudson Bay area. Water is made available at the facility, but no food or heat is provided during the holding period. In October 2018, a batch (mixed) of polar bear fecal materials from an adult female polar bear, her cub, and an adult male polar bear, housed separately from the mother and her cub, was dumped on fresh snow. These bears had been held at the facility for approximately one month. Fecal materials were gathered and placed in two Ziploc bags and brought back to the Churchill Northern Study Center for further separation. Each piece was separately packed in a Whirl-Pak bag and stored at -20°C. Samples were transported to Dalhousie University, Faculty of Agriculture, and stored at -80°C until analysis. A total of nine fecal samples were used for 16s rRNA gene amplicon sequencing. Another wild polar bear fecal sample was found in the Fort Severn area (ON, Canada) in October 2018. The fecal sample was kept at -20°C and shipped to Dalhousie University, Faculty of Agriculture in Jan 2019, and stored at -80°C. No information about the animal(s) was available. Three separate fecal clumps were extracted for 16s rRNA gene amplicon sequencing. To avoid sampling external contaminants, the fecal samples were slightly thawed for one hour then sliced in half and a pea-sized sample (approximately 0.2 g) was taken from the center.

### Captive polar bear monthly fecal sample collection

From March 2018 to September 2020 (two years and seven months), dropped fecal samples were collected monthly from three adult male bears identified as Ganuk, Henry, and Inukshuk, who were housed at the Cochrane Polar Bear habitat in Cochrane, ON, Canada, a fenced 24-acre polar bear habitat with five outdoor enclosures ranging in size from 0.5 to 21 acres with access to the natural environment, including a lake. From March 2019 to September 2020, a fourth captive male polar bear identified as Eddy, who was temporarily relocated from the Aquarium de Quebec (Quebec City, QC, Canada), also joined the monthly fecal sample collection. Background information on the four captive bears, including age, body weight, habitat and nutritional history are described in S8 Table.

To collect the fecal samples, an individually wrapped Puritan® 6" Sterile Standard Cotton Swab (25–806 PC) was inserted into the centre of a fresh fecal deposit, swirled, placed in a 1.5 ml RNA-free microcentrifuge tube containing 1.2 ml RNAlater^TM^ Stabilization Solution (Thermo Fisher Scientific), and stored at -20°C. Fecal samples were transported to Dalhousie University, Faculty of Agriculture, on ice, then stored in -80°C until analysis. A total of 112 fecal samples were collected and analyzed.

### Captive polar bear macroalgae feeding trials

A brown seaweed, *Fucus spiralis*, was collected from the intertidal zones in Churchill (MB, Canada) in October 2018, where polar bears were seen to frequent. The seaweed was packed, frozen at -20° C, shipped to Dalhousie University, Faculty of Agriculture, processed to remove any other species, rinsed and stored at -20° C. Prior to the trials, the seaweed was thawed, bagged in 200 g portions, shipped to the Cochrane Polar Bear Habitat, and stored at -20° C until the commencement of the feeding trials.

Two 28-day feeding trials with similar trial designs were conducted. The first feeding trial occurred from April 9th—May 7th, 2019, and Inukshuk, Henry and Ganuk participated. The second feeding trial took place from April 8th—May 6th, 2020, to increase the number of replicates and enhance statistical relevancy. The second trial included the three male polar bears from the first trial as well as a fourth male, Eddy. During each 28-day feeding trial, each captive polar bear received a pre-weighed daily portion of 200 g (1.7–3.5% of their diet, depending on the individual) of thawed seaweed in addition to their regular diet. Fecal samples were collected from each bear on days 0, 7, 14, 21 and 28 in the individual’s housing facility, using the sample collection procedure described above.

The two seaweed feeding trials were approved and carried out under the direction and guidance of the animal care committee of the Cochrane Polar Bear. Bears’ participation in either seaweed feeding trial was volunteer-based and all bears consumed any food items they were presented, including seaweed, willingly.

### DNA isolation and 16s rRNA gene amplicon sequencing

Fecal samples stored in RNAlater^TM^ were centrifuged at 15,000 x g for 1 min to remove the reagent. All fecal samples were subjected to DNA extraction using the DNeasy PowerSoil Pro Kit (QIAGEN, Germany), following the manufacturer’s instructions, at the Atlantic Research Center for Agricultural Genomics (Faculty of Agriculture, Dalhousie University, NS, Canada). DNA concentration and purity were analyzed using a NanoDrop 2000 (Thermo Scientific, USA). Extracted samples were adjusted to ensure a concentration of DNA between 50 to 100 ng/μL, then sent for 16S rRNA gene amplicon sequencing (2 x 300 bp reads) at the Integrated Microbiome Resource (Dalhousie University, NS, Canada) using Illumina MiSeq technology, as described in Comeau et al. [39]. The following primers were used to target the 438bp V6-V8 hypervariable region: 969F = 5’-ACGCGHNRAACCTTACC-3’ and 1406R = 5’-ACGGGCRGTGWGTRCAA-3’ [39]. The V6-V8 region was chosen due to its increased success in capturing Arctic marine bacterial diversity [40].

Four negative controls were processed concurrently with the samples at the extraction stage and included for sequencing. These controls demonstrated weak or no amplification during the PCR step, leading to either weak or failed sequencing outcomes. This indicated minimal contamination and therefore they were discarded.

### Bioinformatics and statistical analysis

Sequence reads were processed in QIIME 2 (version 2022.4) using the workflow described by Comeau et al. [39], with minor modifications [41]. Paired-end and demultiplexed reads were trimmed for primers using the Cutadapt QIIME 2 plugin [42]. The paired-end reads were joined with VSEARCH [43], and subjected to low-quality filtering. All sequences were then trimmed to 399 bp and denoised using Deblur [44], and any sequence below this threshold was discarded. Sequences were blasted against the Silva reference classifier (silva-138-99-nb) [45], and taxonomy was assigned to amplicon sequence variants (ASVs). Sequence alignment was performed using the MAFFT plugin [46], and an unrooted phylogenetic tree was created using the FastTree plugin [47]. Finally, a rooted tree was created using the midpoint method. All plugins were used with the default settings unless stated otherwise.

Processed data resulting from QIIME 2 were transferred to R (version 4.2.2) for downstream processing using the ‘phyloseq’ ‘qiime2R’ and ‘microbiomeutilities’ packages [48–50]. Mitochondrial and chloroplast 16S sequences, and ASVs with a frequency less than 10 counts were filtered. Samples containing less than 8,164 reads were excluded, resulting in the removal of four samples (Henry-day28-2020, Henry-day7-2020, Henry-April-2018, and Ganuk-Nov-2019). For both alpha and beta diversity, the dataset was rarefied (one-time subsampling) to an even depth at the minimal sample depth of 8,164 reads using the rarefy_even_depth function within the phyloseq package. Alpha diversity of the fecal microbiome, including observed ASVs, Chao1, Shannon, inverse Simpson and Faith’s phylogenetic diversity indices, was calculated using the ‘estimate_richness’ and ‘estimate_pd’ functions within ‘phyloseq.’ Alpha diversity indices were then used to compare wild and captive samples, or variations within the captive population, using one-way analysis of variance (ANOVA), followed by the Tukey-Kramer test if the p-value was less than 0.05, using the MIXED Procedure of SAS (version 9.4) [51]. For the alpha diversity of fecal samples from the captive bear seaweed feeding trials, a similar statistical approach was used, except the analysis was conducted using repeated measures with the variance component structure based on the lowest absolute values of corrected Akaike Information Criterion and Bayesian Information Criterion, compared to other structures including compound symmetry, first-order autoregressive, Toeplitz, and unstructured covariance. The trial period (days 0, 7, 14, 21, and 28 after seaweed consumption) was the main effect, with each individual bear as an experimental unit, and the specific trial (2019 or 2020 trial) as a blocking factor to account for potential effects specific to the trial or year. The residuals met the assumptions of normal distribution for all datasets.

For beta diversity visualization, the Bray-Curtis dissimilarity, or unweighted or weighted UniFrac distance matrix were ordinated using either non-metric multidimensional scaling (NMDS) or principal coordinate analysis (PCoA) methods using the ‘plot_ordination’ function within the ‘phyloseq’ package in R. Following visualization, both permutational multivariate analysis of variance (PERMANOVA) and analysis of similarities (ANOSIM) were used as follow-up statistical tests, using the ‘adonis2’ or ‘anosim’ functions within ‘vegan’ package [52]. To check the assumption of PERMANOVA, homogeneity of group dispersions was calculated using the ‘betadisper’ function in ‘vegan’ package. If PERMANOVA indicated significance (p < 0.05), pairwise PERMANOVA tests were performed using package ‘pairwiseAdonis’ and p-values were adjusted using the Bonferroni method [53]. The Venn diagram was generated with the unrarefied dataset using ‘MicrobiotaProcess’ and ‘VennDiagram’ packages [54,55]. For compositional analysis of fecal microbiome, including relative abundance and differential abundance analyses, unrarefied data were used. Relative abundance was analyzed using the Kruskal-Wallis and Dunn tests due to most datasets violated the assumption of normal distribution. Analysis of compositions of microbiomes with bias correction (ANCOM-BC) was performed on compositions of microbiome for differential abundance analysis using the ‘ANCOMBC’ package in R [56]. ANCOM-BC was selected, as this method considers the compositional nature of microbiome data, thereby correcting this bias and resulting in a lower rate of false positive detection. All data were visualized using the R packages ‘ggplot2’ and ‘ggpubr’ unless stated otherwise [57,58].

## Supporting information

S1 Table Alpha diversity of the fecal microbiome of captive bears in Cochrane compared with wild bears in Churchill and Fort Severn. (DOCX)

S2 Table PERMANOVA, ANOSIM and pairwise PERMANOVA analyses on the fecal microbiome of polar bears from Cochrane (captive), Churchill (wild), and Fort Severn (wild). (DOCX)

S3 Table Relative abundance (%) of fecal microbiome at the phylum level comparing captive and wild populations. (DOCX)

S4 Table Alpha diversity of the fecal microbiome of captive polar bears with the exclusion of bear Eddy. (DOCX)

S5 Table PERMANOVA and ANOSIM analyses based on Bray-Curtis distance matrix of captive polar bear fecal microbiome by individual differences, season, and year, with the exclusion of bear Eddy. (DOCX)

S6 Table Alpha diversity of the fecal microbiome of captive bears fed seaweed. (DOCX)

S7 Table PERMANOVA and ANOSIM analyses of fecal microbiome of captive polar bears fed seaweed. (DOCX)

S8 Table Background information of the four captive polar bears housed at the Cochrane Polar Bear Habitat (Cochrane, ON, Canada). (DOCX)

S1 Fig Observed ASVs, Chao1 and Faith’s phylogenetic diversity comparing the fecal microbiome of captive to wild bears. ANOVA results indicated no significant differences with p-values of 0.833 for observed ASVs, 0.513 for Chao1, and 0.610 for Faith’s phylogenetic diversity.
(TIF)

S2 Fig Beta diversity of the fecal microbiome of captive polar bears in the Cochrane habitat and wild polar bears in Churchill and Fort Severn, demonstrated in NMDS plots ordinated by Bray-Curtis (a) and unweighted UniFrac (b) distance matrices. Stress values were 0.191 and 0.156, respectively.
(TIF)

S3 Fig Fecal microbial composition analysis with ANCOM-BC at the family level: A comparison between the captive and wild populations. (TIF)

S4 Fig Beta diversity of captive polar bear fecal microbiome, with the exclusion of bear Eddy, demonstrated by Bray-Curtis distance matrix in PCoA plots: Effects of **a)** individual differences, **b)** season, **c)** year.
(TIF)

S5 Fig Beta diversity plots of the fecal microbiome of captive bears fed seaweed. (TIF)

S6 Fig Fecal microbial composition analysis of captive polar bears fed seaweed after 7, 14, 21, and 28 days compared to day 0: ANCOM-BC at the genus level. (TIF)
