# Supplementary material for: Distinct fecal microbiome between wild and habitat-housed captive polar bears (Ursus maritimus): Impacts of captivity and dietary shifts
Source: PLoS One. 2024 Nov 20;19(11):e0311518. doi: 10.1371/journal.pone.0311518 (PMC11578516; doi:10.1371/journal.pone.0311518)
Supplement: S1 Table — (DOCX) [file pone.0311518.s001.docx]

S1 Table. Alpha diversity of the fecal microbiome of captive bears in Cochrane compared with wild bears in Churchill and Fort Severn.

|  | **Observed ASVs** | **Chao1** | **Shannon** | **Inverse Simpson** | **Faith’s phylogenetic diversity** |
| --- | --- | --- | --- | --- | --- |
| Cochrane (Captive) | 134.6 ± 39.2 | 181.9 ± 57.2 | 2.8 ± 0.4 ^a^ | 8.5 ± 3.9 ^a^ | 18.3 ± 5.2 |
| Churchill (Wild) | 143.9 ± 53.4 | 185.9 ± 59.1 | 2.5 ± 0.6 ^a^ | 6.8 ± 4.0 ^ab^ | 20.4 ± 5.3 |
| Fort Severn (Wild) | 96.7 ± 26.5 | 124.4 ± 38.9 | 1.4 ± 0.2 ^b^ | 2.2 ± 0.3 ^b^ | 15.2 ± 2.7 |
| **ANOVA p-value** | 0.207 | 0.222 | < 0.001*** | 0.0011** | 0.278 |

Mean ± SD. Different superscripts in the same column indicate statistical significance (p < 0.05).
