# Supplementary material for: Distinct fecal microbiome between wild and habitat-housed captive polar bears (Ursus maritimus): Impacts of captivity and dietary shifts
Source: PLoS One. 2024 Nov 20;19(11):e0311518. doi: 10.1371/journal.pone.0311518 (PMC11578516; doi:10.1371/journal.pone.0311518)
Supplement: S2 Table — (DOCX) [file pone.0311518.s002.docx]

S2 Table. PERMANOVA, ANOSIM and pairwise PERMANOVA analyses on the fecal microbiome of polar bears from Cochrane (captive), Churchill (wild), and Fort Severn (wild).

|  | **PERMANOVA** | | |  | **ANOSIM** | |
| --- | --- | --- | --- | --- | --- | --- |
|  | p-value | R^2^ | Homogeneity of group dispersions |  | p-value | R |
| Bray-Curtis | < 0.001*** | 0.184 | 0.134 |  | < 0.001*** | 0.781 |
| Unweighted UniFrac | < 0.001*** | 0.115 | < 0.001*** |  | < 0.001*** | 0.892 |
|  | **Pairwise PERMANOVA** | | | | | |
| Bray-Curtis | Cochrane (captive) ^a^ Churchill (wild) ^b^ Fort Severn (wild) ^b^ | | | | | |
| Unweighted UniFrac | Cochrane (captive) ^a^ Churchill (wild) ^b^ Fort Severn (wild) ^b^ | | | | | |

Different superscripts in the same row indicate statistical significance (p < 0.05).
