# Supplementary material for: Distinct fecal microbiome between wild and habitat-housed captive polar bears (Ursus maritimus): Impacts of captivity and dietary shifts
Source: PLoS One. 2024 Nov 20;19(11):e0311518. doi: 10.1371/journal.pone.0311518 (PMC11578516; doi:10.1371/journal.pone.0311518)
Supplement: S3 Table — (DOCX) [file pone.0311518.s003.docx]

S3 Table. Relative abundance (%) of fecal microbiome at the phylum level comparing captive and wild populations.

| **Relative abundance (%)** | **Captive (n=135)** | **Wild (n=12)** | **p-value** |
| --- | --- | --- | --- |
| *Firmicutes* | 69.4±17.0 | 51.3±25.1 | 0.0138* |
| *Proteobacteria* | 19.9±14.6 | 34.3±24.1 | 0.0608 |
| *Bacteroidota* | 8.7±15.6 | 6.7±14.1 | 0.9944 |
| *Actinobacteriota* | 1.1±2.6 | 7.5±8.5 | >0.001** |
| *Fusobacteriota* | 0.811±2.294 | 0.0±0.0 | >0.001** |
| *Desulfobacterota* | 0.040±0.119 | 0.267±0.915 | 0.1554 |
| *Campilobacterota* | 0.027±0.064 | 0.009±0.031 | 0.0142* |
| *Unclassified bacteria* | 0.009±0.018 | 0.0±0.0 | 0.0025** |
| *Bdellovibrionota* | 0.0±0.0 | 0.005+0.010 | >0.001** |
| *Verrucomicrobiota* | 0.0±0.0 | 0.009+0.020 | >0.001** |
