# Supplementary material for: Distinct fecal microbiome between wild and habitat-housed captive polar bears (Ursus maritimus): Impacts of captivity and dietary shifts
Source: PLoS One. 2024 Nov 20;19(11):e0311518. doi: 10.1371/journal.pone.0311518 (PMC11578516; doi:10.1371/journal.pone.0311518)
Supplement: S4 Table — (DOCX) [file pone.0311518.s004.docx]

S4 Table. Alpha diversity of the fecal microbiome of captive polar bears with the exclusion of bear Eddy.

|  | **By individual differences** | | |  |  |
| --- | --- | --- | --- | --- | --- |
|  | Ganuk | Henry | Inukshuk |  | **ANOVA p-value** |
| Observed ASVs | 146.9 ± 38.4 ^a^ | 141.3 ± 42.7 ^ab^ | 117.8 ± 36.8 ^b^ |  | 0.021* |
| Chao1 | 205.6 ± 60.3 ^a^ | 186.4 ± 52.0 ^ab^ | 160.1 ± 53.0 ^b^ |  | 0.014* |
| Shannon | 2.9 ± 0.5 | 2.8 ± 0.4 | 2.7 ± 0.5 |  | 0.381 |
| Inverse Simpson | 9.0 ± 3.1 | 8.8 ± 3.1 | 8.0 ± 3.8 |  | 0.547 |
| Faith’s phylogenetic diversity | 19.9 ± 5.1 ^a^ | 19.3 ± 5.7 ^a^ | 15.5 ± 4.2 ^b^ |  | 0.004** |
|  |  |  |  |  |  |
|  | **By season** | | | |  |
|  | Spring | Summer | Fall | Winter | **ANOVA p-value** |
| Observed ASVs | 148.5 ± 23.5 | 135.7 ± 39.5 | 117.9 ± 34.2 | 139.0 ± 56.2 | 0.171 |
| Chao1 | 208.3 ± 38.0 | 187.6 ± 55.3 | 161.3 ± 56.5 | 178.5 ± 71.6 | 0.116 |
| Shannon | 2.7 ± 0.5 | 2.8 ± 0.4 | 2.8 ± 0.4 | 2.9 ± 0.6 | 0.851 |
| Inverse Simpson | 6.9 ± 2.7 | 9.4 ± 3.3 | 9.2 ± 3.3 | 8.5 ± 3.7 | 0.087 |
| Faith’s phylogenetic diversity | 20.8 ± 3.2 ^a^ | 18.5 ± 5.1 ^ab^ | 15.3 ± 4.8 ^b^ | 18.3 ± 6.6 ^ab^ | 0.026* |
|  |  |  |  |  |  |
|  | **By year** | | |  |  |
|  | 2018 | 2019 | 2020 |  | **ANOVA p-value** |
| Observed ASVs | 129.1 ± 37.4 | 132.6± 40.6 | 145.5 ± 44.4 |  | 0.336 |
| Chao1 | 184.6 ± 55 | 175.5 ± 56.1 | 194.1 ± 63.3 |  | 0.515 |
| Shannon | 2.7 ± 0.4 | 2.9 ± 0.4 | 2.7 ± 0.5 |  | 0.228 |
| Inverse Simpson | 7.9 ± 3.3 | 9.5 ± 3.1 | 8.3 ± 3.6 |  | 0.178 |
| Faith’s phylogenetic diversity | 16.8± 4.7 | 18.3 ± 5.2 | 19.8 ± 5.8 |  | 0.135 |

Mean ± SD. Different superscripts in the same row indicate statistical significance (p < 0.05).
