# Supplementary material for: Distinct fecal microbiome between wild and habitat-housed captive polar bears (Ursus maritimus): Impacts of captivity and dietary shifts
Source: PLoS One. 2024 Nov 20;19(11):e0311518. doi: 10.1371/journal.pone.0311518 (PMC11578516; doi:10.1371/journal.pone.0311518)
Supplement: S5 Table — (DOCX) [file pone.0311518.s005.docx]

S5 Table. PERMANOVA and ANOSIM analyses based on Bray-Curtis distance matrix of captive polar bear fecal microbiome by individual differences, season, and year, with the exclusion of bear Eddy.

|  | **PERMANOVA on Bray-Curtis** | | |  | **ANOSIM on Bray-Curtis** | |
| --- | --- | --- | --- | --- | --- | --- |
|  | p-value | R^2^ | Homogeneity of group dispersions |  | p-value | R |
| By individual differences | 0.002** | 0.059 | 0.263 |  | 0.002** | 0.076 |
| By season | < 0.001*** | 0.126 | 0.226 |  | < 0.001*** | 0.165 |
| By year | 0.015* | 0.048 | 0.795 |  | 0.009** | 0.055 |
| Pairwise PERMANOVA | Spring ^b^ Summer ^c^ Fall ^a^ Winter ^ab^ | | | |  |  |

Different superscripts in the same row indicate statistical significance (p < 0.05).
