# Supplementary material for: Distinct fecal microbiome between wild and habitat-housed captive polar bears (Ursus maritimus): Impacts of captivity and dietary shifts
Source: PLoS One. 2024 Nov 20;19(11):e0311518. doi: 10.1371/journal.pone.0311518 (PMC11578516; doi:10.1371/journal.pone.0311518)
Supplement: S6 Table — (DOCX) [file pone.0311518.s006.docx]

S6 Table. Alpha diversity of the fecal microbiome of captive bears fed seaweed.

|  | **Observed** | **Chao1** | **Shannon** | **Inverse Simpson** | **Faith’s phylogenetic diversity** |
| --- | --- | --- | --- | --- | --- |
| **Trial period** |  |  |  |  |  |
| Day 0 | 142.0 ± 31.4 | 195.8 ± 40.3 | 2.9 ± 0.3 ^ab^ | 8.7 ± 3.5 | 21.1 ± 4.0 |
| Day 7 | 144.2 ± 31.7 | 198.5 ± 55.3 | 3.1 ± 0.20 ^a^ | 11.0 ± 2.0 | 19.8 ± 3.7 |
| Day 14 | 143.3 ± 23.4 | 213.3 ± 45.2 | 2.5 ± 0.5 ^b^ | 5.7 ± 2.7 | 19.1 ± 3.9 |
| Day 21 | 117.7 ± 20.4 | 137.2 ± 30.3 | 2.8 ± 0.3 ^ab^ | 9.0 ± 3.8 | 16.9 ± 4.1 |
| Day 28 | 113.2 ± 47.7 | 166.0 ± 80.8 | 2.8 ± 0.3 ^ab^ | 9.4 ± 3.7 | 14.1 ± 6.1 |
|  |  |  |  |  |  |
| ANOVA p-value |  |  |  |  |  |
| Trial period | 0.323 | 0.137 | 0.041* | 0.148 | 0.126 |
| Year (2019 or 2020 trial)^1^ | 0.510 | 0.945 | 0.161 | 0.491 | 0.273 |

^1^ Year was used as a blocking factor

Mean ± SD. Different superscripts in the same column indicate statistical significance (p < 0.05).
