# Supplementary material for: Distinct fecal microbiome between wild and habitat-housed captive polar bears (Ursus maritimus): Impacts of captivity and dietary shifts
Source: PLoS One. 2024 Nov 20;19(11):e0311518. doi: 10.1371/journal.pone.0311518 (PMC11578516; doi:10.1371/journal.pone.0311518)
Supplement: S7 Table — (DOCX) [file pone.0311518.s007.docx]

S7 Table. PERMANOVA and ANOSIM analyses of the fecal microbiome of captive polar bears fed seaweed.

|  | **PERMANOVA** | | |  | **ANOSIM** | |
| --- | --- | --- | --- | --- | --- | --- |
|  | p-value | R^2^ | Homogeneity of group dispersions |  | p-value | R |
| Bray-Curtis | 0.170 | 0.173 | 0.868 |  | 0.237 | 0.036 |
| Unweighted UniFrac | 0.237 | 0.126 | 0.290 |  | 0.280 | 0.033 |
| Weighted UniFrac | 0.163 | 0.178 | 0.593 |  | 0.176 | 0.048 |
