# Supplementary material for: Distinct fecal microbiome between wild and habitat-housed captive polar bears (Ursus maritimus): Impacts of captivity and dietary shifts
Source: PLoS One. 2024 Nov 20;19(11):e0311518. doi: 10.1371/journal.pone.0311518 (PMC11578516; doi:10.1371/journal.pone.0311518)
Supplement: S8 Table — (DOCX) [file pone.0311518.s008.docx]

S8 Table. Background information of the four captive polar bears housed at the Cochrane Polar Bear Habitat (Cochrane, ON, Canada).

| **Bear** | **Sex** | **Age class during data collection period** | **Age during data collection period** | **Body weight (lb)** | **History** | **Diet (per day)** |
| --- | --- | --- | --- | --- | --- | --- |
| Ganuk | Male | Sub-adult to adult | 8-10 | ~1000 | Born in a Quebec Zoo and arrived at the Habitat in 2012 | 4.0-4.5 kg mackerel, 0.5 kg moose meat, 0.1kg seal oil, 1.0 kg produce in summer/fall |
| Henry | Male | Sub-adult | 4-6 | ~700-900 | Born in an Australian zoo and arrived at the Habitat in 2015 | 4.0-5.0 kg mackerel, 0.5-0.7 kg moose meat, 0.2 kg seal meat, 0.25-0.5 kg seal oil, 1.0 kg produce in summer/fall |
| Inukshuk | Male | Adult | 15-17 | ~930 | Born in the wild near Fort Severn, ON, Canada and arrived at the Habitat in 2016 | 4.0 kg mackerel, 0.5-0.8 kg moose meat, 0.3 kg seal meat, 1.0 kg produce in summer/fall |
| Eddy | Male | Adult | 19-20 | ~1000 | Born in a Quebec Zoo and arrived at the Habitat in 2018. Deceased in 2021 due to kidney disease | 4.5-6 kg mackerel, 0.2-0.3 kg moose meat, 0.1 kg seal oil, 1.0 kg produce in summer/fall |

*Produce includes items such as lettuce, carrot, sweet potato, apple

**Occasionally, bears received treats such as 1-2 teaspoons of peanut butter, 0.5 - 1.0 kg salmon, pumpkin, watermelon, cantaloupe
