# Supplementary figures and images for: Distinct fecal microbiome between wild and habitat-housed captive polar bears (Ursus maritimus): Impacts of captivity and dietary shifts
Source: PLoS One. 2024 Nov 20;19(11):e0311518. doi: 10.1371/journal.pone.0311518 (PMC11578516; doi:10.1371/journal.pone.0311518)

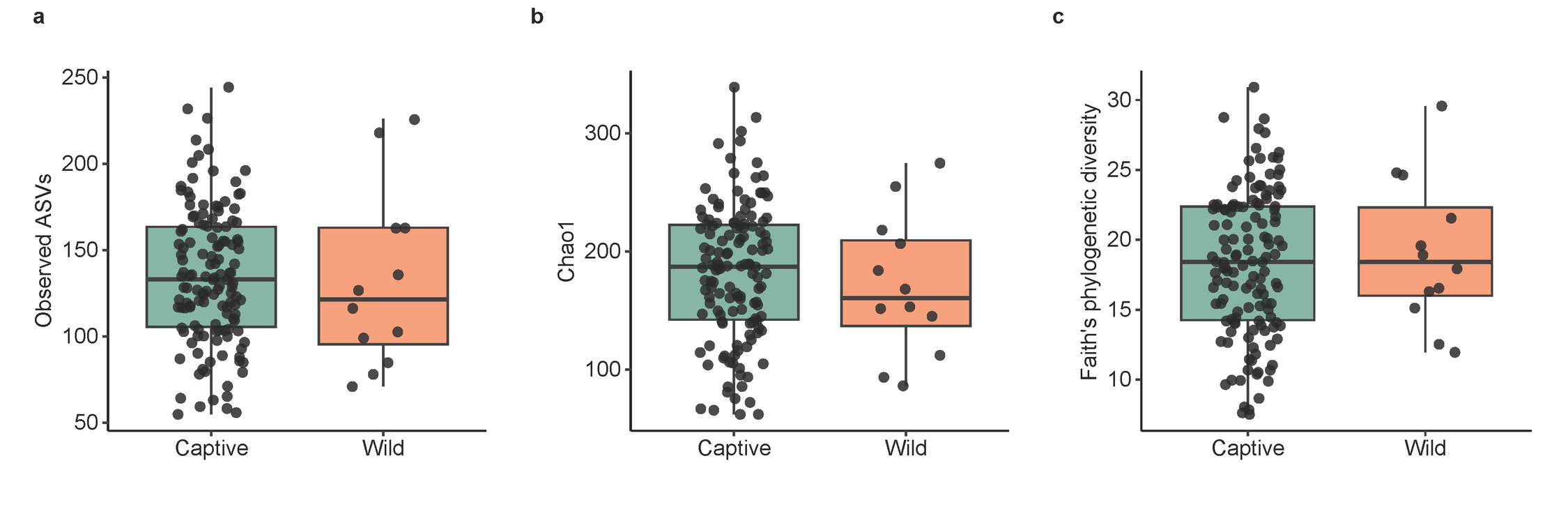

Supplement: S1 Fig — ANOVA results indicated no significant differences with p-values of 0.833 for observed ASVs, 0.513 for Chao1, and 0.610 for Faith’s phylogenetic diversity. (TIF) [file pone.0311518.s009.tif]

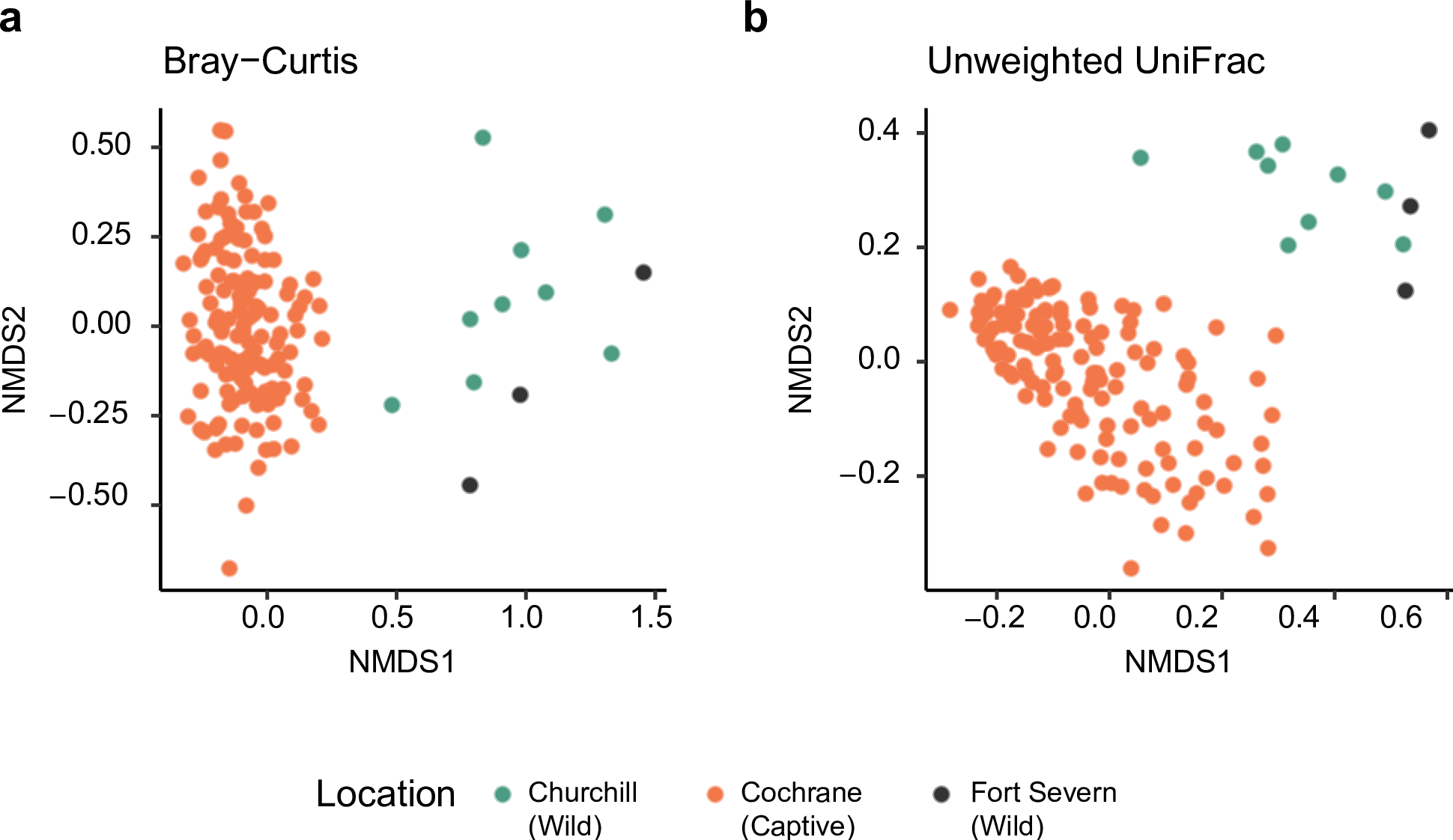

Supplement: S2 Fig — Beta diversity of the fecal microbiome of captive polar bears in the Cochrane habitat and wild polar bears in Churchill and Fort Severn, demonstrated in NMDS plots ordinated by Bray-Curtis (a) and unweighted UniFrac (b) distance matrices. Stress values were 0.191 and 0.156, respectively. (TIF) [file pone.0311518.s010.tif]

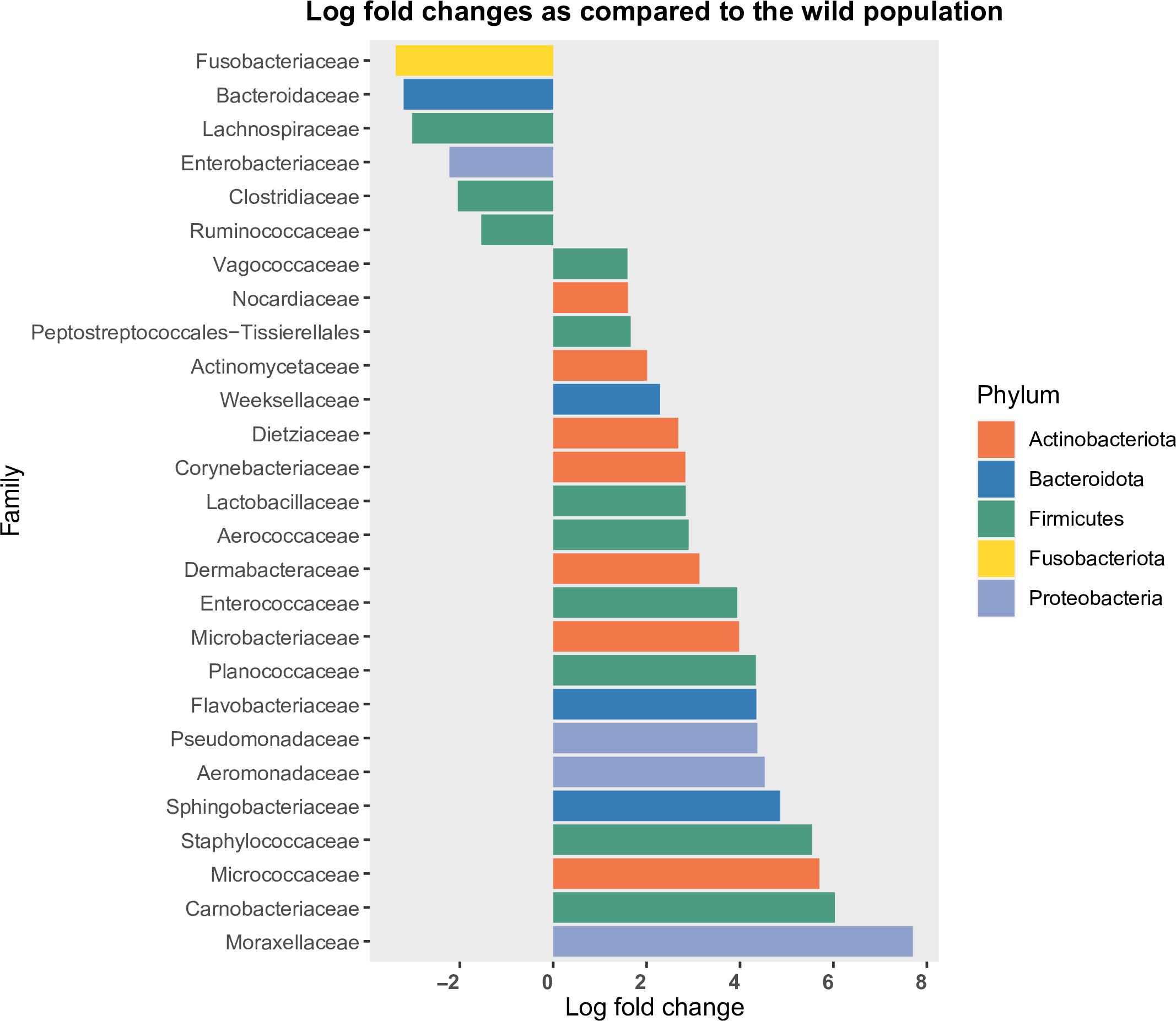

Supplement: S3 Fig — (TIF) [file pone.0311518.s011.tif]

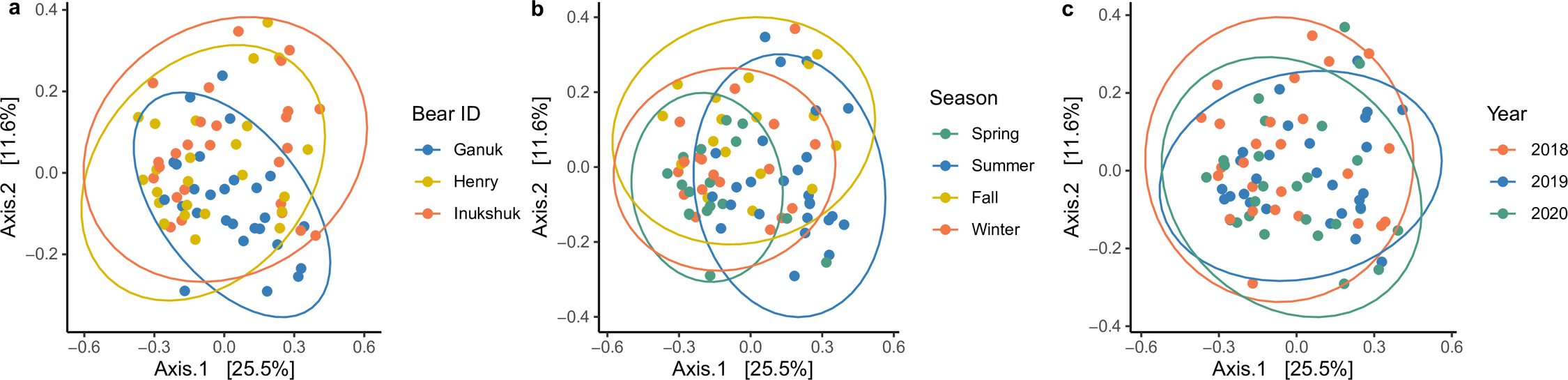

Supplement: S4 Fig — Beta diversity of captive polar bear fecal microbiome, with the exclusion of bear Eddy, demonstrated by Bray-Curtis distance matrix in PCoA plots: Effects of a) individual differences, b) season, c) year. (TIF) [file pone.0311518.s012.tif]

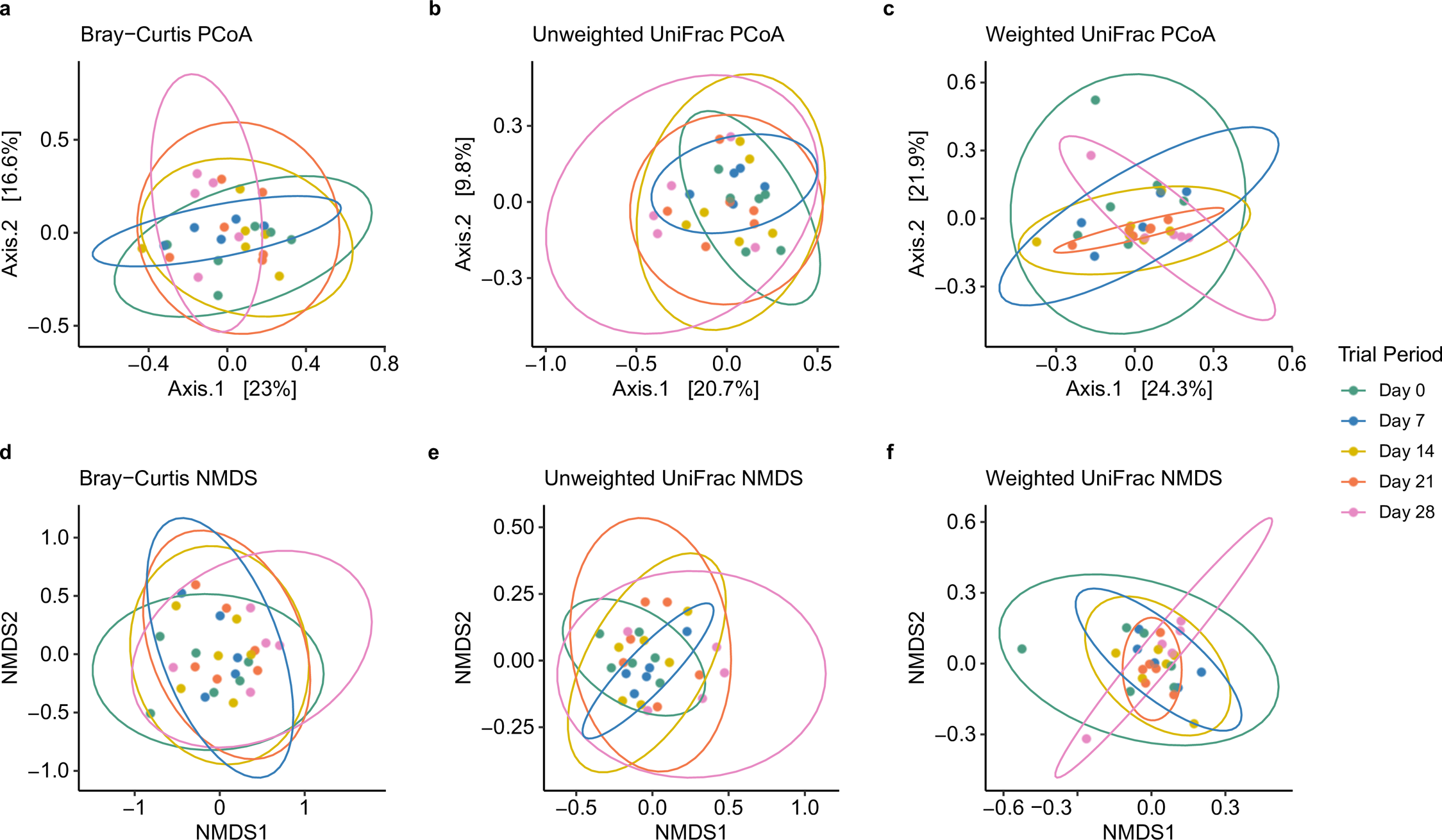

Supplement: S5 Fig — (TIF) [file pone.0311518.s013.tif]

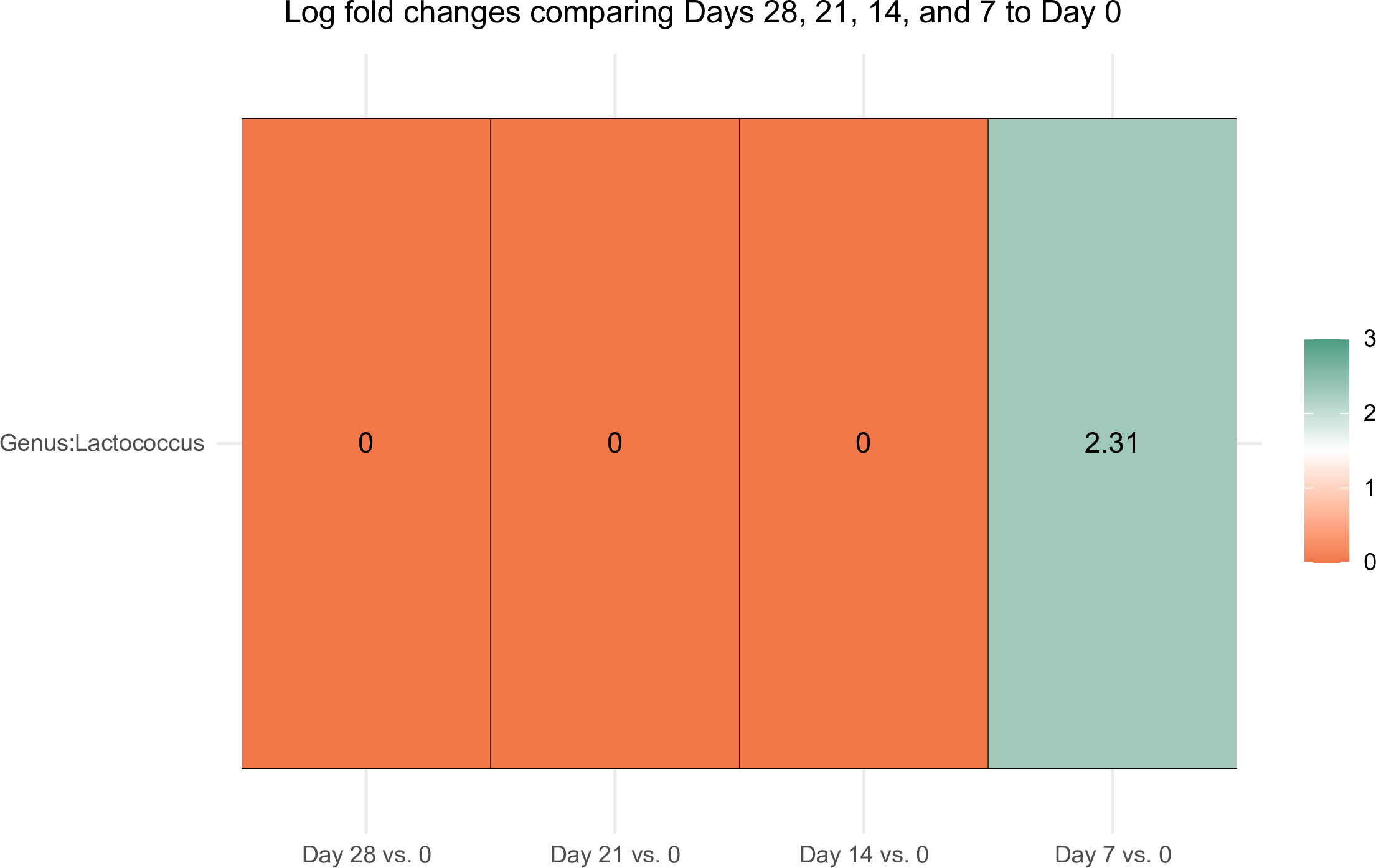

Supplement: S6 Fig — (TIF) [file pone.0311518.s014.tif]
